# Don’t Flush the Yuck Factor

**DOI:** 10.1289/ehp.0800542

**Published:** 2009-03

**Authors:** John Manuel

**Affiliations:** Freelance Writer, Durham, North Carolina, E-mail: john.manuel@gte.net

I enjoyed Charles Schmidt’s original and informative article on the “Yuck Factor” (Schmidt 2008). This phenomenon has come into play in my attempts to decide on whether to purchase a low-flow toilet for my home. My city is currently offering rebates on low-flow toilets, but only on the least consumptive models (1.6 gallons per flush). I have read on web sites and heard from friends that these models do not quite do the job. The details are important to me. If it is a question of not flushing gobs of toilet paper down, I can deal with that. But if these toilets leave stains on (or product in) the bowl, my family will not be happy. My discussions with city officials and friends who have installed these toilets invariably break down when I press for details. The “Yuck Factor” reigns supreme. The end result may be that I do not purchase a low-flow toilet.

## References

[b1-ehp-117-a99] Schmidt CW (2008). The yuck factor: when disgust meets discovery. Environ Health Perspect.

